# Polydopamine (PDA) coatings with endothelial vascular growth factor (VEGF) immobilization inhibiting neointimal formation post zinc (zn) wire implantation in rat aortas

**DOI:** 10.1186/s40824-023-00423-5

**Published:** 2023-09-04

**Authors:** Jiayin Fu, Qiongjun Zhu, Zhezhe Chen, Jing Zhao, Shaofei Wu, Meng Zhao, Shihui Xu, Dongwu Lai, Guosheng Fu, Wenbin Zhang

**Affiliations:** Key Laboratory of Cardiovascular Intervention and Regenerative Medicine of Zhejiang Province, Department of Cardiology, Sir Run Run Shaw Hospital, Zhejiang University, Hangzhou, 310016 China

**Keywords:** Polydopamine (PDA), Endothelial vascular growth factor (VEGF), Neointimal formation, Zinc (zn), Degradation

## Abstract

**Background:**

Bioresorbable stents are designed to provide temporary mechanical support to the coronary arteries and then slowly degrade in vivo to avoid chronic inflammation. Zinc (Zn) is a promising material for bioresorbable stents; However, it can cause inflammation and neointimal formation after being implanted into blood vessels.

**Methods:**

To improve biocompatibility of Zn, we first coated it with polydopamine (PDA), followed by immobilization of endothelial vascular growth factor (VEGF) onto the PDA coatings. Adhesion, proliferation, and phenotype maintenance of endothelial cells (ECs) on the coated Zn were evaluated in vitro. Then, a wire aortic implantation model in rats mimicking endovascular stent implantation in humans was used to assess vascular responses to the coated Zn wires in vivo. Thrombosis in aortas post Zn wire implantation, degradation of Zn wires in vivo, neointimal formation surrounding Zn wires, and macrophage infiltration and extracellular matrix (ECM) remodeling in the neointimas were examined.

**Results:**

In vitro data showed that the PDA-coated Zn encouraged EC adhesion, spreading, proliferation, and phenotype maintenance on its surfaces. VEGF functionalization on PDA coatings further enhanced the biocompatibility of Zn to ECs. Implantation of PDA-coated Zn wires into rat aortas didn’t cause thrombosis and showed a faster blood flow than pure Zn or the Zn wires coated with VEGF alone. In addition, the PDA coating didn’t affect the degradation of Zn wires in vivo. Besides, the PDA-coated Zn wires reduced neointimal formation, increased EC coverage, decreased macrophage infiltration, and declined aggrecan accumulation in ECM. VEGF immobilization onto PDA coatings didn’t cause thrombosis and affect Zn degradation in vivo as well, and further increased the endothelization percentage as compared to PDA coating alone, thus resulting in thinner neointimas.

**Conclusion:**

These results indicate that PDA coatings with VEGF immobilization would be a promising approach to functionalize Zn surfaces to increase biocompatibility, reduce inflammation, and inhibit neointimal formation after Zn implantation in vivo.

**Graphical Abstract:**

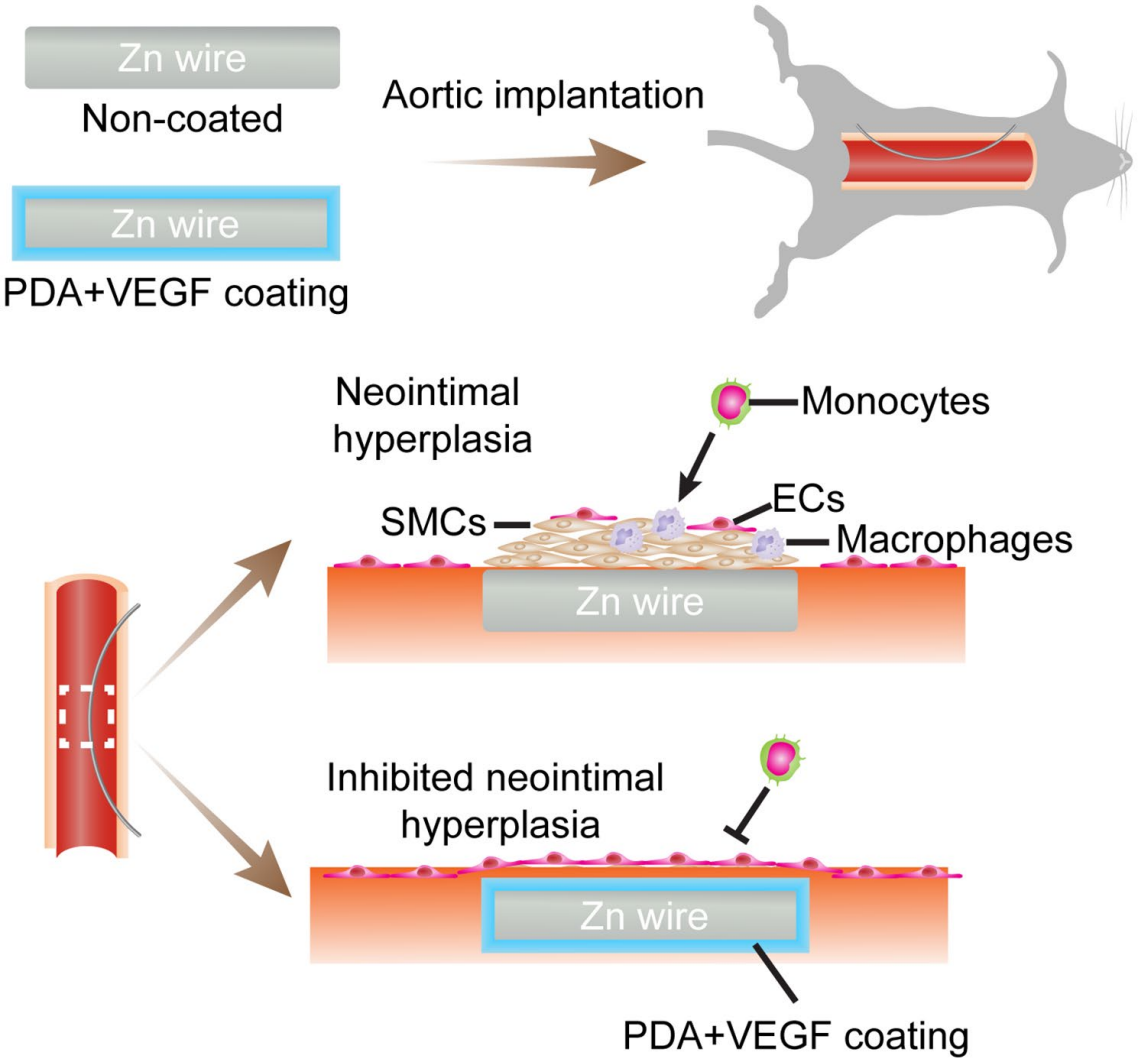

## Introduction

Endovascular stent placement post coronary angioplasty is known to prevent acute vascular cloture and chronic artery narrowing arising from vascular remodeling. Unfortunately, non-degradable stents can remain in blood vessels for lengthy periods and trigger chronic inflammation via immune cells as foreign bodies. The inflammation then stimulates the local vascular smooth muscle cell (SMC) proliferation and eventually leads to in-stent restenosis [1]. Bioresorbable stents are then designed to provide temporary mechanical support to the coronary arteries and degrade slowly in vivo to avoid chronic inflammation caused by non-degradable stents. Zinc (Zn) is a promising bioresorbable stent material as it can degrade slowly to provide sufficient mechanical support to blood vessels before vascular remodeling is finished and then quickly after complete vascular remodeling [2–6]. Furthermore, Zn is essential for numerous enzymatic reactions in vivo and has unique cardioprotective roles against atherosclerosis [7]. Mechanical strength of Zn is also much higher than bioresorbable polymers, such as poly-L-lactic acid [3]. Nonetheless, Zn can induce inflammation and neointimal formation in blood vessels post implantation [8–12]. Meanwhile, the excessive Zn^2+^ released when Zn degrades can hinder cell adhesion and mobility [13]. Therefore, biocompatibility of Zn still needs to be improved to increase the efficacy of the bioresorbable stents and hence the clinical outcome of patients implanted with the stents.

To overcome the challenge of poor cell adhesion and growth on Zn substrates, surface modifications are usually required. Manipulation of surface topography on Zn substrates is one strategy. It has been shown that topography in microscales on Zn plates can reduce macrophage inflammatory polarization and enhance adhesion and differentiation of bone cells as compared to topography in nanoscales [14]. Another study shows that zinc phosphate coatings on Zn surfaces can improve the viability, adhesion and proliferation of the seeded cells [15]. Although the in vitro data show promising results, further studies are needed to validate the efficacy of these coatings in vivo.

Dopamine can spontaneously polymerize under alkaline conditions to deposit a thin and adherent polydopamine (PDA) coating onto various substrates [16]. Catechol and quinone functional groups in PDA coatings make nucleophiles can be covalently coupled to them as ad-layers [17]. In addition, the coating method is simple, which just needs immersing objects in an aqueous dopamine solution for dip-coating. Meanwhile, dopamine coating alone has been shown to increase biocompatibility of implanted materials [18]. PDA can also eliminate reactive oxygen species (ROS) as radical scavengers because of abundant phenol groups [19]. Due to these unique properties, PDA is widely used for surface functionalization of medical implants. For example, hyaluronic acid and PDA coatings are fabricated on 316 L stainless steels or cobalt-chromium to improve their hemocompatibility and reduce the activation of macrophages on their surfaces [20–22]. A similar strategy is also used to modify surfaces of poly(dimethylsiloxane) (PDMS) or poly(urethane) (PU) substrates, which shows an enhanced hemocompatibility and anti-inflammation effects as compared to those unmodified PDMS or PU [21, 23]. Vascular endothelial growth factors (VEGFs) immobilized on of PDA coatings increase endothelial cell (EC) attachment, viability, and proliferation onto 316 L stainless steels and titanium substrates [24, 25]. PDA coating constructed on titanium substrates is also used to immobilize heparin/poly L-lysine nanoparticles to enhance hemocompatibility and anti-inflammatory properties of the titanium substrates [26, 27]. In addition, PDA coatings selectively inhibit SMC proliferation while improve EC proliferation [28]. However, most of the above results were obtained through in vitro studies, and the effects of the PDA-based coating in vivo are lacking. Moreover, whether PDA-based coating can improve biocompatibility of Zn-based stents remains unknown.

**Fig. 1:**
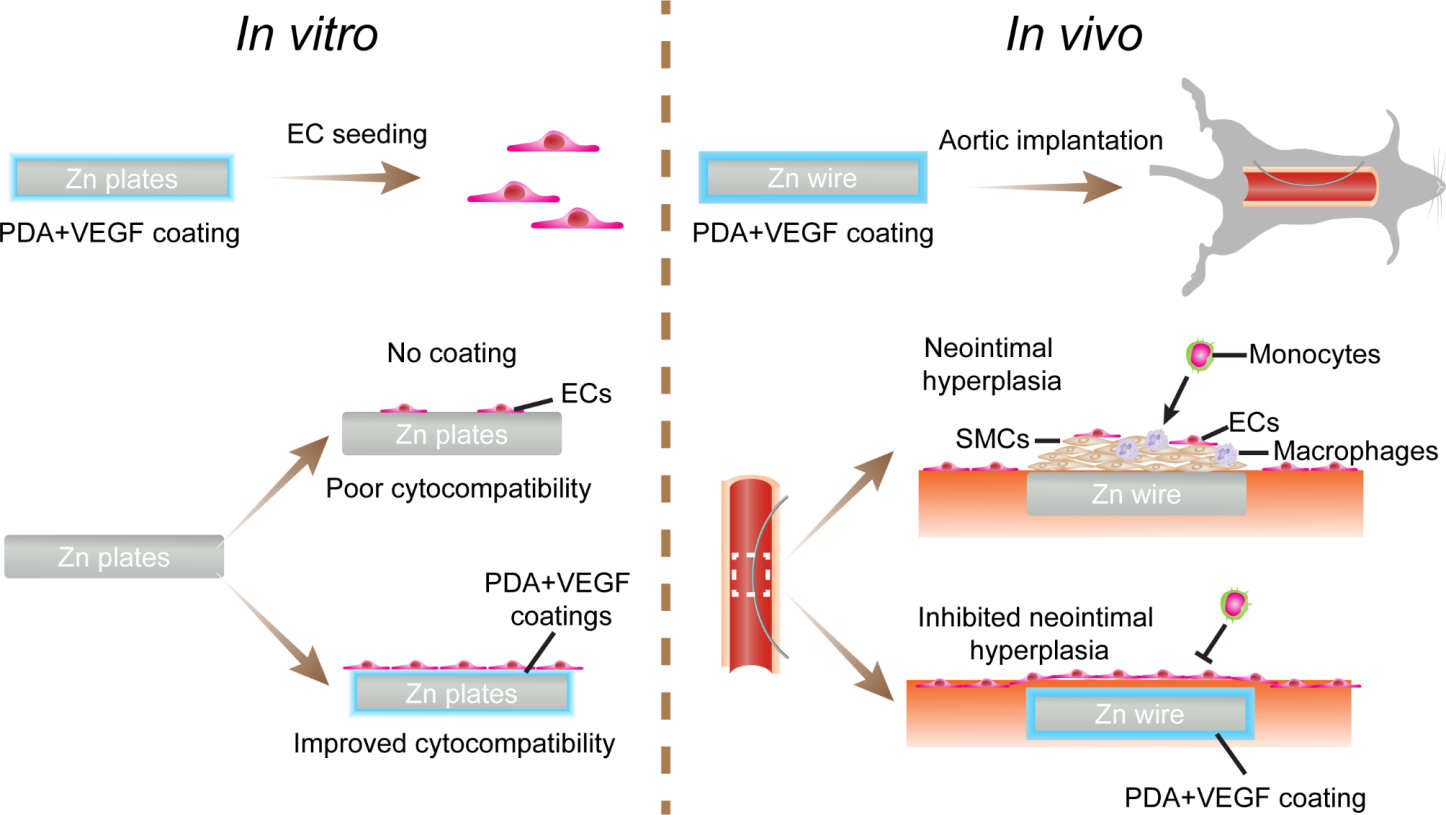
Schematic illustrations of the effects of PDA and VEGF coating on cytocompatibility of ECs on Zn surfaces in vitro and neointimal formation surrounding Zn wires in vivo

Since VEGF can accelerate reendothelialization after medical device implantation [29] and PDA can inhibit SMC proliferation while improve EC proliferation [28], we assume that VEGF immobilization on PDA-coated Zn substrates, new materials for bioabsorbable stents, can promote reendothelialization and at the same inhibit intimal hyperplasia after Zn implantation in vivo (Fig. 1). To validate this hypothesis, we first coated the Zn substrates with PDA and then immobilized VEGF onto the PDA coatings. The adhesion, proliferation, and phenotype maintenance of ECs on the coated Zn were evaluated. Then, a wire aortic implantation model in rats mimicking endovascular stent implantation in humans was used to assess vascular responses to the coated Zn wires in vivo. Thrombosis in aortas post Zn wire implantation, degradation of Zn wires in vivo, neointimal formation surrounding Zn wires, and macrophage infiltration and extracellular matrix (ECM) remodeling in the neointimas were examined.

## Methods

### Surface functionalization

Zn wires (0.25 mm in diameter and 15 mm in length, Goodfellow, UK) or Zn sheets (0.05 mm in thickness, 10 mm × 10 mm, square, Goodfellow, UK) were immersed in dopamine hydrochloride solutions (Sigma, 1 mg/mL in 10 mM Tris buffer, pH 8.5) at room temperature overnight for dopamine polymerization. After that, the Zn wires or sheets were washed with deionized (DI) water to remove unbound dopamine. The polydopamine (PDA)-coated wires or sheets were further immersed in VEGF^165^ protein solutions (MCE, China, 0.5 mg/mL in 10 mM Tris buffer, pH 8.5) at 37 ℃ overnight for VEGF^165^ deposition. The Zn wires or sheets that were coated with PDA or VEGF^165^ alone were used as a comparison. The human recombinant VEGF^165^ was used for cell culture in vitro, and the rat recombinant VEGF^165^ was used for animal experiment in vivo.

### Surface characterization

Zn wires (0.25 mm in diameter and 15 mm in length) or Zn sheets (0.05 mm in thickness, 10 mm × 10 mm, square) were used in this experiment. To visualize proteins immobilized on surfaces of Zn wires or sheets, rhodamine B isothiocyanate (RBITC) modified bovine serum albumin (RBITC-BSA, Zhong Ke Chen Yu Life Science, Beijing, China) was used as a model molecule. The diluted RBITC-BSA solution (0.57 mg/mL in 10 mM Tris buffer, pH 8.5) was added to the PDA-coated Zn wires or sheets and incubated with them overnight at 37 ℃. After washing three times with phosphate buffered saline (PBS), the Zn sheets and wires were observed with fluorescence microscopes (IX73, Olympus, Japan) and confocal fluorescence microscope (FV1200, Olympus, Japan), respectively. BCA Protein Assay Kit (Pierce, Thermo Scientific) was used to quantify the protein adsorbed onto the surfaces of the Zn substrates. Three different samples (n = 3) for each group were quantified. To reveal the microstructures of the surfaces of the coated Zn substrates, the coated Zn sheets were washed with PBS, and fixed in 2.5% glutaraldehyde at room temperature for 30 min. After dehydration in gradient ethanol, samples were coated with gold and observed under a field-emitting scanning electron microscope (SEM, COXEM, Korea). To track the release of the adsorbed RBITC-BSA from the surfaces of the Zn materials, the coated Zn sheets were incubated in PBS at 37 ℃ for 7 days. At the designated timepoints, the sheets were read under IVIS® Lumina LT In Vivo Imaging System (IVIS, PerkinElmer) and the fluorescence intensity of three different samples (n = 3) for each group were quantified.

### Cell culture

Human umbilical vein endothelial cells (HUVECs, Meisen, China) were maintained in endothelial cell growth medium (ECM, ScienCell, USA) at 37 ℃ in a humidified atmosphere containing 5% CO_2_ with medium replaced every day. Upon 80% confluence, HUVECs were dissociated from tissue culture flasks with trypsin and seeded onto coated Zn sheets. Cells with a passage number between 2 and 8 were used in this study.

### Cell adhesion tests

Zn sheets (0.05 mm in thickness, 10 mm × 10 mm, square) were used for all the cell experiments. HUVECs were labeled with CMFDA (CellTracker™ Green, Invitrogen) as manuals for visualization. After that, the labeled HUVECs were seeded on different Zn substrates. 12 h later, cells were imaged with a fluorescence microscope. Three different samples (n = 3) for each group were quantified.

### Cell spreading tests

After 7 days of culture on different Zn surfaces, HUVECs were washed with PBS, fixed with 4% paraformaldehyde (PFA, HaoKe Biotechnology, Hangzhou, China), and permeabilized with 0.1% Triton X-100 for 10 minutes respectively at room temperature. After that, the cells were stained with rhodamine phalloidin (Invitrogen). Nuclei were counterstained with 4’, 6-diamidino-2-phenylindole (DAPI, Sigma). The stained cells were imaged with a fluorescence microscope and the cell covered areas were quantified with the ImageJ software. 20 different cells (n = 20) for each group were quantified.

### Cell proliferation assays

Cell proliferation was evaluated with cell counting kit-8 (CCK-8, Dojindo, Japan) as instructions. Briefly, after 3, 5, and 7 days of culture on different Zn surfaces, HUVECs were incubated in ECM containing 10% CCK-8 at 37 ℃ for 2 h. Then, the OD value of the supernatants was measured with a microplate reader at a wavelength of 450 nm. At the same time, cells were stained with rhodamine phalloidin and DAPI as aforementioned and imaged with a fluorescence microscope for quality evaluation of cell proliferation. Three different samples (n = 3) for each group were tested.

### NO probe detection

The NO levels in cells were detected with a fluorescence NO probe, 3-Amino,4-aminomethyl-2’,7’-difluorescein diacetate (DAF-FM DA, Beyotime Biotechnology, China) as manuals. Briefly, 5µM DAF-FM DA was incubated with HUVECs after 7 days of culture on different Zn substrates for 20 min. After that, cells were gently washed with PBS to remove free probes and imaged with a fluorescence microscope. Three different samples (n = 3) for each group were quantified.

### Immunocytochemistry

HUVECs were cultured on different Zn substrates for 7 days. After that, cells were washed with PBS and fixed in 4% PFA solution for 10 min at room temperature. After being permeabilized with 0.1% Triton X-100 for 10 min at room temperature, cells were incubated with the following primary antibodies in 5% bovine serum albumin (BSA) solution for 1 h at room temperature: endothelial nitric oxide synthase antibody (eNOS, ab5589, Abcam) and von Willebrand Factor antibody (VWF, sc-365,712, Santa Cruz Biotechnology). Then, the samples were washed with PBS again and incubated with goat anti-rabbit secondary antibody (A-11,037, Invitrogen) for 1 h at room temperature. Nuclei were counterstained with DAPI. The stained samples were then imaged with a fluorescence microscope. Three different samples (n = 3) for each group were quantified.

### Aortic implantation

Zn wires (0.25 mm in diameter and 15 mm in length) were used for all the animal experiments. All the animal experiments were approved by the Chinese Institutional Animal Care and Use Committee at Sir Run Run Shaw Hospital, School of Medicine, Zhejiang University. Male young SD rats (age: 6–8 weeks, body weight: 300–400 g, Laboratory Animal Center in Zhejiang Province, Hangzhou, China) were used for aortic implantation. Four different groups of samples were implanted into animals, including the Zn group (Zn wires without any coating), the VEGF group (Zn wires coated with VEGF alone), the PDA group (Zn wires coated with PDA alone), and the PDA + VEGF group (Zn wires coated with PDA and VEGF). 5 animals were used for each group (n = 5). Totally, 20 rats were used in this study. Aortic implantation was performed as described previously [5, 12]. Briefly, a midline incision was made on the abdomens of rats to expose the abdominal aorta. The aorta was separated from the inferior vena cava and the blood flow in the aorta was blocked with a microvascular clamp. Zn wires were then inserted into the aorta followed by the removal of the microvascular clamp to recover the blood flow. The surgical site was closed with sutures lastly. No anticoagulation or antiplatelet treatments were administrated pre- or post-operatively.

### Patency monitoring with ultrasound

VisualSonics high resolution ultrasound imaging system (Vevo 3100, FUJIFILM) was used to monitor blood flow in the abdominal aortas 4 weeks after implantation. Animals were anesthetized by isoflurane inhalation (5% for induction, and then 1.5% for maintenance). The abdomens were opened to expose the aortas and ultrasound gels were applied to the aortas. Cross section images of the aortas in B mode, color mode, and PW mode were acquired. The diameters of the aortas were evaluated with B mode images. The patience of the abdominal aorta was determined by color mode images and parameters regarding the blood flow, such as velocity time integral (VTI), mean velocity and mean gradient, were measured by PW mode images.

### Micro-computed tomography (micro-CT) and SEM scanning

Before implantation, the pure Zn wires and the coated Zn wires were scanned with high resolution micro-CT (Skyscan 1275, Bruker, Belgium) to record their baseline volume. 1 month after implantation, the rats were sacrificed, and the implanted Zn wires were dissected out for micro-CT scanning again to evaluate the degradation of the Zn wires. The Zn wires were scanned with micro-CT at a resolution of 12 μm/pixel. Volume reduction, volume and surface areas of each wire was calculated from the 3-dimensional (3D) reconstruction of the scanned images. To characterize the surface erosion of the implanted Zn wires, the tissues surrounding the wires were carefully peeled off and the wires were dehydrated in gradient ethanol. After being coated with gold, the samples were observed under field-emitting SEM (Nova Nano 450, Czech).

### Chemical staining

The aortas implanted with Zn wires were fixed in 4% PFA at 4 °C overnight. The fixed samples were further soaked in 30% sucrose solution (Sigma, China) at 4 °C for 24 h and embedded into the optimal cutting temperature compound (OCT, Sakura), snap-frozen at -80 °C, and cryo-sectioned at 10 μm in thickness. Slides were then stained with hematoxylin and eosin (H&E), Masson’s trichrome (MTC), Elastic Verhoeff-Van Gieson (EVG) and alizarin red S (ARS). All the stained slides were captured with an inverted microscope (IX73, Olympus, Japan). Five different tissue sections (n = 5) from five different rats for each group were quantified.

### Terminal deoxynucleotidyl transferase-mediated dUTP nick end labeling (TUNEL) and reactive oxygen species (ROS) staining

To evaluate cell apoptosis surrounding the Zn wires, the tissue sections were stained with TUNEL using In Situ Cell Death Detection Kit (Roche) as manuals. To assess ROS levels, the samples were stained with an ROS detection kit (BestBio, China) as instructed. Nuclei were counterstained with DAPI. The stained slides were then imaged with a fluorescence microscope (IX73, Olympus, Japan). Five different tissue sections (n = 5) from five different rats for each group were quantified.

### Immunofluorescence staining

Samples were washed with PBS three times and then blocked with 5% BSA solution for 30 min, followed by incubation with the primary antibodies diluted in 5% BSA solution overnight at 4 °C: eNOS (ab5589, Abcam), alpha smooth muscle actin antibody (αSMA, ab7817, Abcam), CD68 (MCA341GA, BioRad), Cyclin D1 (A19038, ABclonal), Aggrecan (13880-1-AP, Proteintech), and CD206 (ab64693, Abcam). After that, samples were washed with PBS three times and incubated with goat anti-rabbit secondary antibody or goat anti-mouse antibody for 1 h at room temperature. Nuclei were counterstained with DAPI. Tissue sections without primary antibody incubation were used as negative controls. The stained samples were observed with an inverted microscope (IX73, Olympus, Japan). Five different tissue sections (n = 5) from five different rats for each group were quantified. Specifically, for CD68^+^ and CD206^+^ cell quantification, two different tissue sections from the same sample were stained with CD68 and CD206 antibodies, respectively. The numbers of the CD68^+^ and CD206^+^ cells and the ratio of CD206^+^ cells to CD68^+^ were then quantified. Five sets of tissues sections (n = 5) from five different rats for each group were quantified.

### Statistics

All data were presented as mean ± standard deviation. Each test had at least three replicates and was repeated three times independently. One-way ANOVA followed by Turkey’s post-Hoc test was used to analyze the data. P < 0.05 was considered statistically significant.

## Results

### Coating construction on Zn surfaces

**Fig. 2:**
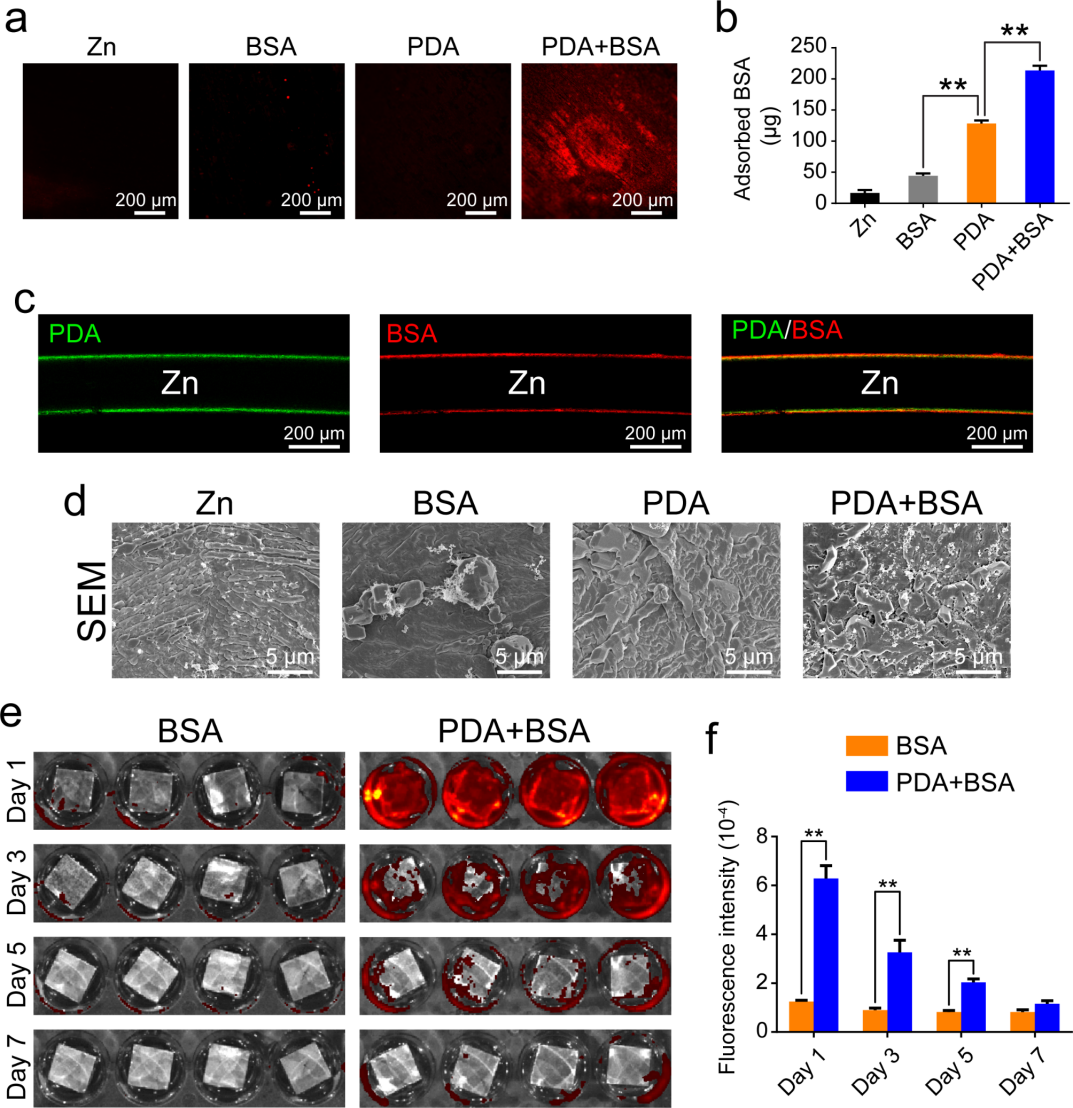
Characterization of PDA-based coatings. (**a**) The deposition of RBITC-labeled BSA on different Zn surfaces. (**b**) The quantification of BSA immobilized on different Zn surfaces. (**c**) Confocal images of Zn wires coated with PDA and RBITC-labeled BSA. (**d**) SEM images of different Zn surfaces. (**e**) Fluorescence images of RBITC-labeled BSA immobilized on non-coated Zn surfaces or on Zn surfaces coated with PDA by IVIS from day 1 to day 7. (**f**) Quantification of fluorescence intensity on non-coated Zn surfaces or on Zn surfaces coated with PDA after RBITC-labeled BSA coating

Dopamine polymerizes spontaneously in alkaline conditions to form a PDA coating on substrate surfaces, which has the ability to immobilize proteins through reaction between amine groups of the proteins and the PDA-coated substrates [16, 17]. BSA was used as a model protein to study protein deposition on the PDA-coated Zn surfaces. To visualize the BSA, RBITC labeled BSA was used in this study. As shown in Fig. 2a, BSA could not be adsorbed onto the surfaces of Zn. However, when the surfaces of Zn were pretreated with PDA, BSA could well deposit on the surfaces, as evidenced by the strong red fluorescence. The deposited BSA on Zn surfaces were further quantified with the BCA Protein Assay Kit. The BSA on the Zn surfaces pretreated with PDA was significantly higher than that on the pure Zn surfaces (Fig. 2b). Although there was no protein coated on the Zn surfaces in the PDA group, the test also showed a positive result. This is probably due to the amine groups of the PDA which can also reduce Cu^2+^ to Cu^1+^ to induce a color change of the reaction, similarly to the amine groups of the proteins. Confocal fluorescence microscope observation further validated the bi-layered coatings: one PDA layer, which exhibited green autofluorescence; the other BSA layer, which showed red fluorescence (Fig. 2c). SEM examination demonstrated that the surfaces of pure Zn and PDA-treated Zn were relatively smoother than those of the BSA and PDA + BSA coated Zn (Fig. 2d). The PDA + BSA group had rougher surfaces than the BSA group, suggesting a higher level of BSA deposition. Stability of the coatings were assessed by incubating the coated Zn plates in PBS at 37 ℃ for 7 days, and fluorescence of the residual BSA was measured. After 1 day of PBS incubation, BSA on the pure Zn surfaces was almost invisible, while the Zn plates in the PDA + BSA group still showed strong fluorescence (Fig. 2e). With the time of incubation, there was a gradual reduction of the fluorescence on the surfaces of PDA-coated Zn plates, which, however, was still stronger than that on the surfaces of pure Zn plates from day 1 to day 7 (Fig. 2f).

### Adhesion and proliferation of HUVECs on the PDA and VEGF-coated Zn surfaces

**Fig. 3:**
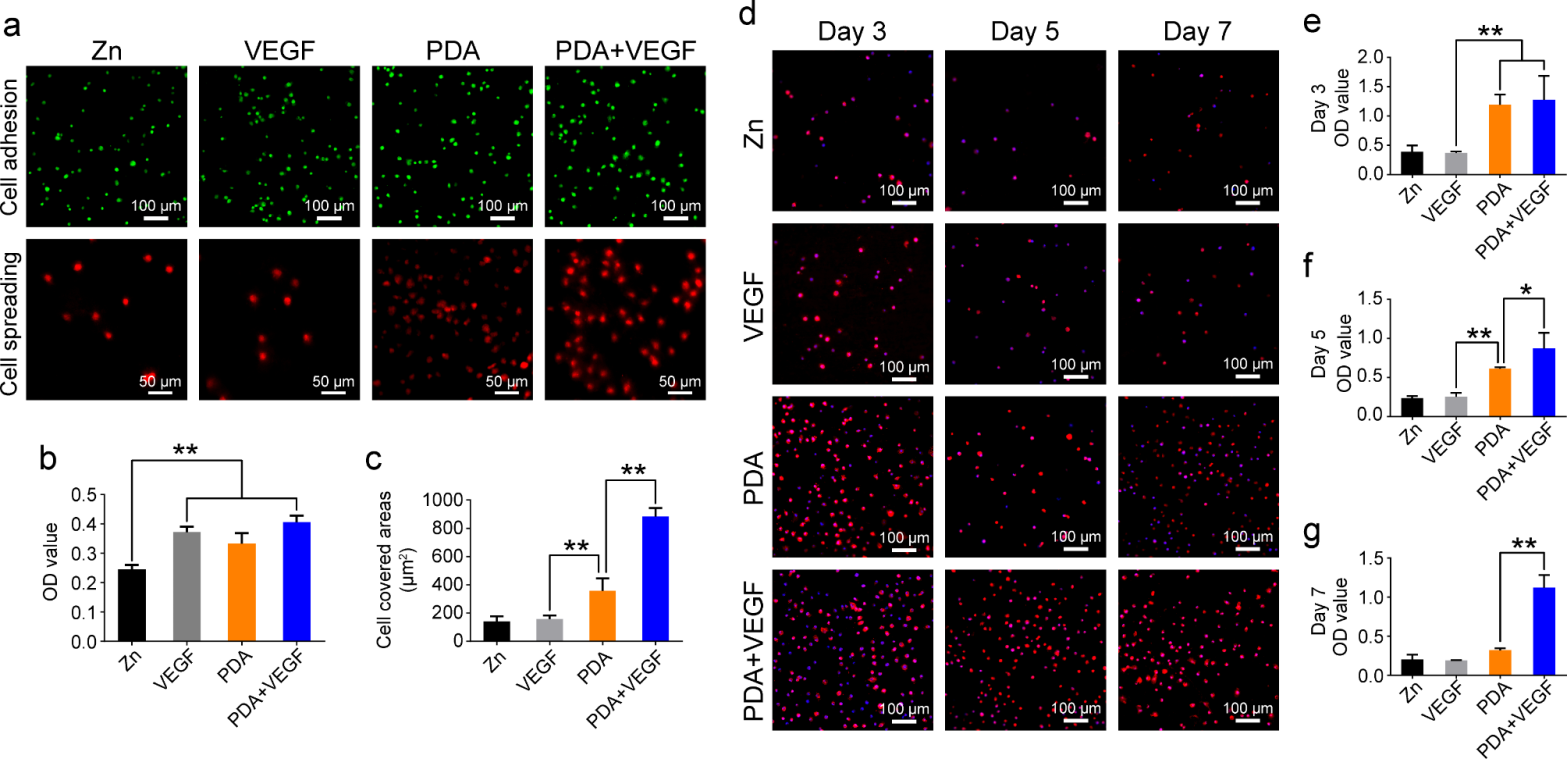
The adhesion and proliferation of HUVECs on different Zn surfaces. (**a**) The adhesion and spreading of HUVECs on different Zn surfaces. (**b**) The CCK-8 results of HUVECs adhered on different Zn surfaces 24 h after cell seeding. (**c**) Quantification of cell covered areas of HUVECs cultured on different Zn surfaces for 7 days. (**d**) The proliferation of HUVECs cultured on different Zn surfaces for 7 days. The CCK-8 results of HUVECs adhered on different Zn surfaces for 3 days (**e**), 5 days (**f**), and 7 days (**g**). * indicates p < 0.05, compared between two groups. ** indicates p < 0.01, compared between two groups

After characterizing PDA-based coatings, we next evaluated behaviors of HUVECs on different Zn surfaces. To facilitate the growth of the HUVECs, VEGF was coated on PDA-treated Zn surfaces. Figure 3a showed that there was no difference in cell adhesion on the VEGF-, PDA-, or PDA + VEGF-coated Zn surfaces 12 h after cell seeding, while there was less cell adhesion on the pure Zn surfaces. Quantification of adhered cells also confirmed this result (Fig. 3b). After 7 days of culture, the HUVECs on the Zn surfaces coated with PDA and VEGF spread well with flat morphologies. However, the HUVECs on the pure Zn surfaces and the Zn surfaces coated with VEGF alone still showed round morphologies. The PDA coating also favored cell spreading but was less effective than the PDA and VEGF coating (Fig. 3a). The PDA group had higher cell covered areas than the VEGF group did, whereas the VEGF plus PDA coating further increased the cell covered areas (Fig. 3c). The HUVECs on the pure Zn and VEGF-coated Zn surfaces had relatively low proliferation capacities from day 3 to day 5 (Fig. 3d-g), indicating an unfavorable microenvironment for HUVECs. The PDA coating improved the biocompatibility of Zn surfaces, as evidenced by the increased cell proliferation from day 3 to day 5. However, cell proliferation decreased on day 7. In contrast, the PDA coating combined with VEGF maintained the proliferation of HUVECs at a relatively high level (Fig. 3d-g). These results indicate that PDA and VEGF coating greatly enhance the biocompatibility of Zn surfaces.

### Phenotype maintenance of HUVECs on PDA and VEGF-coated Zn surfaces

**Fig. 4:**
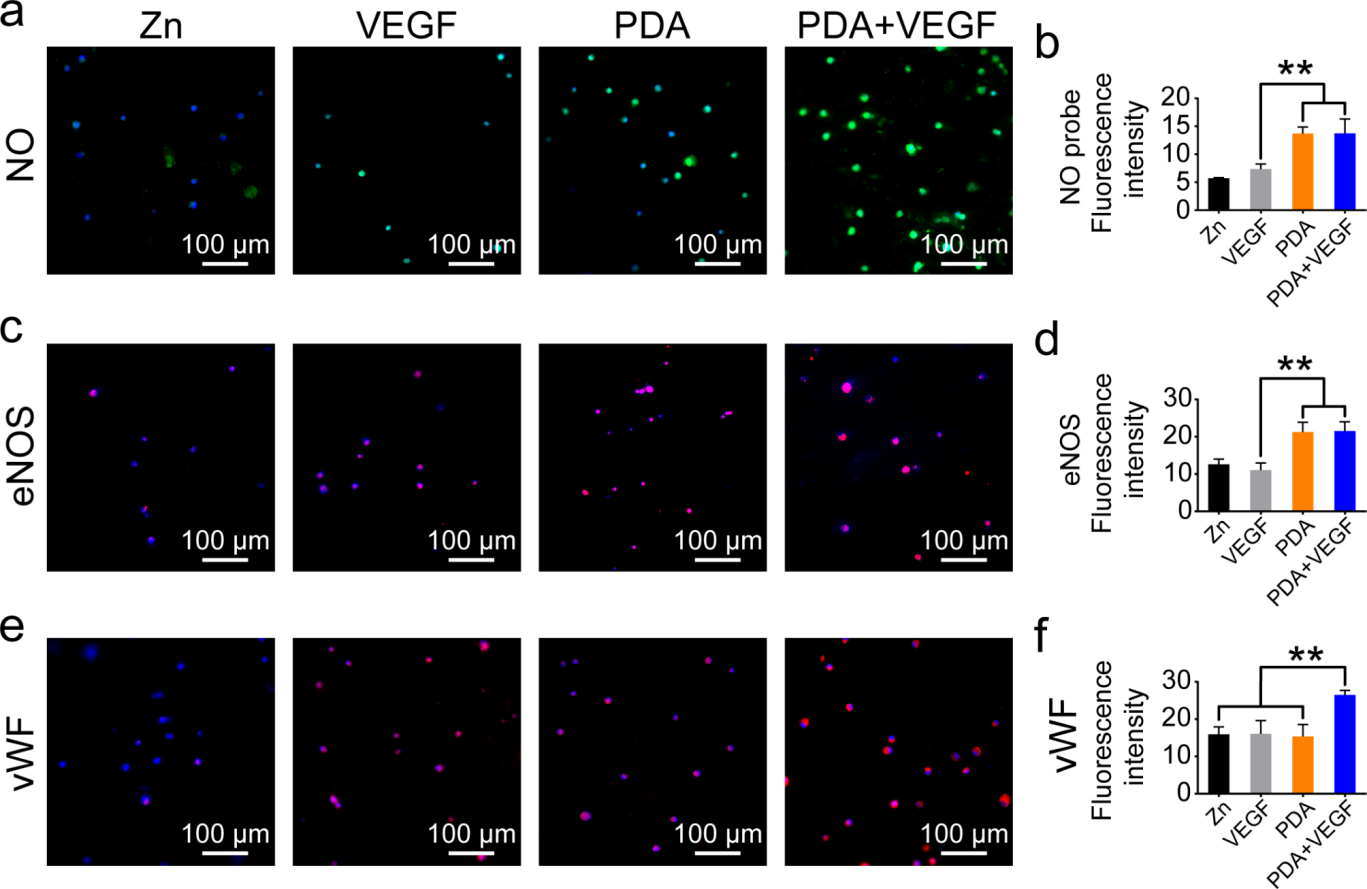
The phenotype maintenance of HUVECs on different Zn surfaces. (**a**) NO levels of HUVECs cultured on different Zn surfaces for 7 days. (**b**) Quantification of fluorescence intensity of NO probes. (**c**) The eNOS staining of HUVECs cultured on different Zn surfaces for 7 days. (**d**) Quantification of fluorescence intensity of eNOS staining. (**e**) The VWF staining of HUVECs cultured on different Zn surfaces for 7 days. (**d**) Quantification of fluorescence intensity of VWF staining. * indicates p < 0.05, compared between two groups. ** indicates p < 0.01, compared between two groups

eNOS is constitutively expressed in endothelial cells (ECs), which is responsible for NO synthesis. To evaluate the phenotypes of the HUVECs on different Zn surfaces, the levels of NO and eNOS expression in cells were detected. As shown in Fig. 4a **and c**, the HUVECs on both PDA- and PDA + VEGF-coated Zn surfaces had higher levels of NO and eNOS than those on the pure Zn surfaces and VEGF-coated Zn surfaces. Quantification of fluorescence intensity of NO and eNOS confirmed these results (Fig. 4b **and d**). Meanwhile, there was no difference in expression of NO and eNOS between the PDA group and the PDA + VEGF group. VWF is another biomarker of endothelial cells. The expression of VWF in the HUVECs on the PDA-coated Zn surfaces was comparable to those on the pure Zn surfaces and VEGF-coated Zn surfaces, which, however, was lower than the PDA + VEGF group (Fig. 4e **and f**). These results imply that PDA coating alone could dramatically improve the phenotype maintenance of HUVECs on Zn surfaces, and VEGF further enhanced cellular functions of HUVECs.

### Patency of aortas post PDA and VEGF-coated Zn wire implantation

**Fig. 5:**
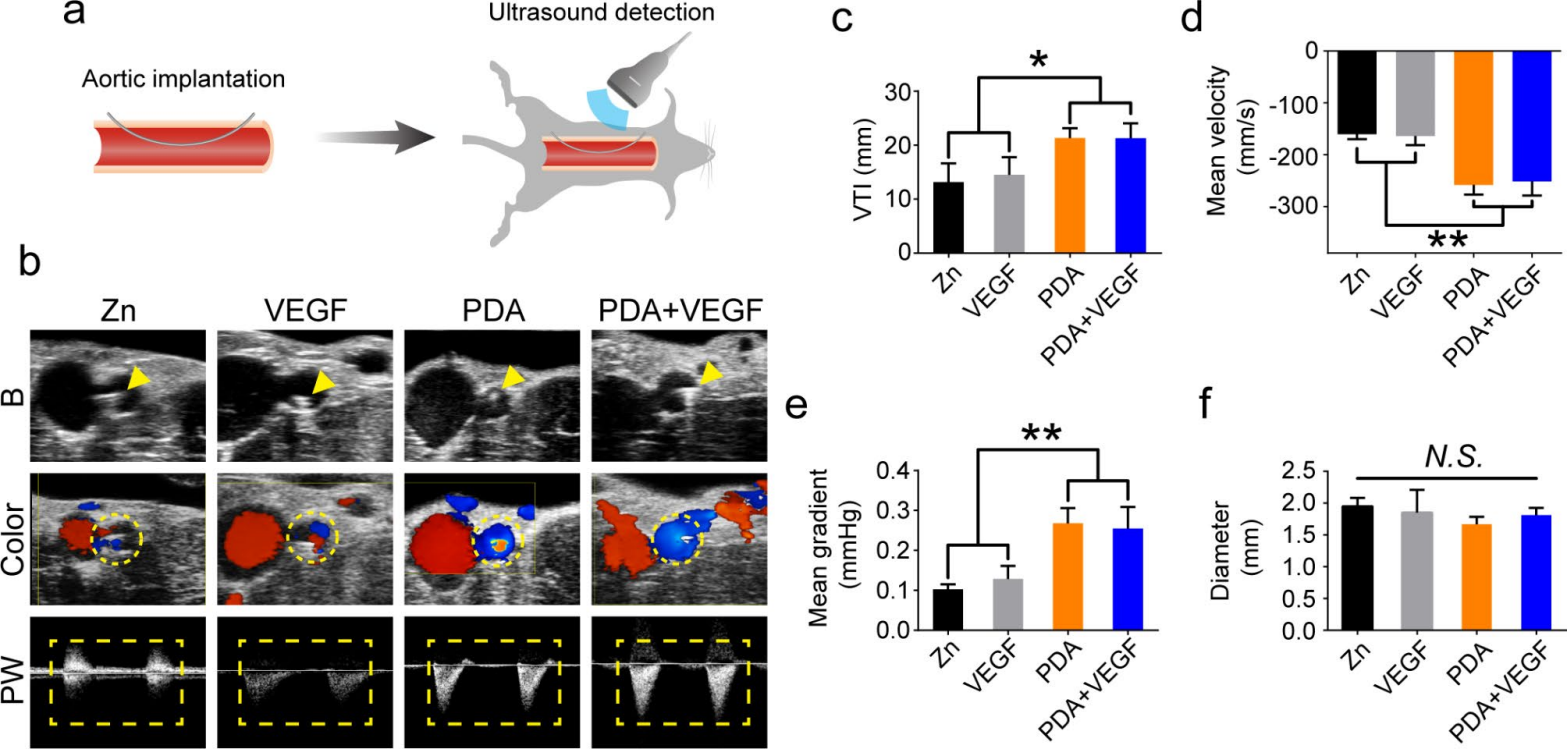
Ultrasound imaging of aortas of rats implanted with different Zn wires. (**a**) Schematic illustrations of the aortic implantation model and ultrasound imaging. (**b**) B mode, Color mode, and PW mode images of the rat aortas implanted with different Zn wires. The arrow heads indicate the implanted Zn wires. The dashed circles indicate the blood flow in the aortas implanted with the Zn wires. The dashed squares indicate the peaks in the PW mode images. VTI (**c**), mean velocity (**d**), and mean gradient (**e**) of the blood flow in the aortas implanted with the different Zn wires. (**f**) Diameter of the aortas implanted with the different Zn wires. * indicates p < 0.05, compared between two groups. ** indicates p < 0.01, compared between two groups

After verification of the efficacy of PDA and VEGF coating in promoting cell growth and functions on Zn surfaces in vitro, vascular responses to this coating were further evaluated in vivo using an aortic implantation model (Fig. 5a). The implanted Zn wires could be well identified with ultrasound in B mode (arrow heads, Fig. 5b). Color mode showed a greater blood flow in the PDA and PDA + VEGF groups compared to the pure Zn and VEGF groups, though the aortas implanted with Zn wires in all the groups were patent (dashed circles, Fig. 5b). Accordingly, PW mode showed high and sharp peaks in the PDA and PDA + VEGF groups, whereas low and flat peaks were observed in the pure Zn and VEGF groups (dashed squares, Fig. 5b). Detailed analysis of the peaks in PW mode revealed that compared to the pure Zn and VEGF groups, the PDA and PDA + VEGF groups had longer VTI, faster mean velocity, and higher gradient (Fig. 5c-e). Nonetheless, there was no difference in diameter of the aortas implanted with Zn wires among different groups (Fig. 5f).

### Degradation of PDA and VEGF-coated Zn wires in vivo

**Fig. 6:**
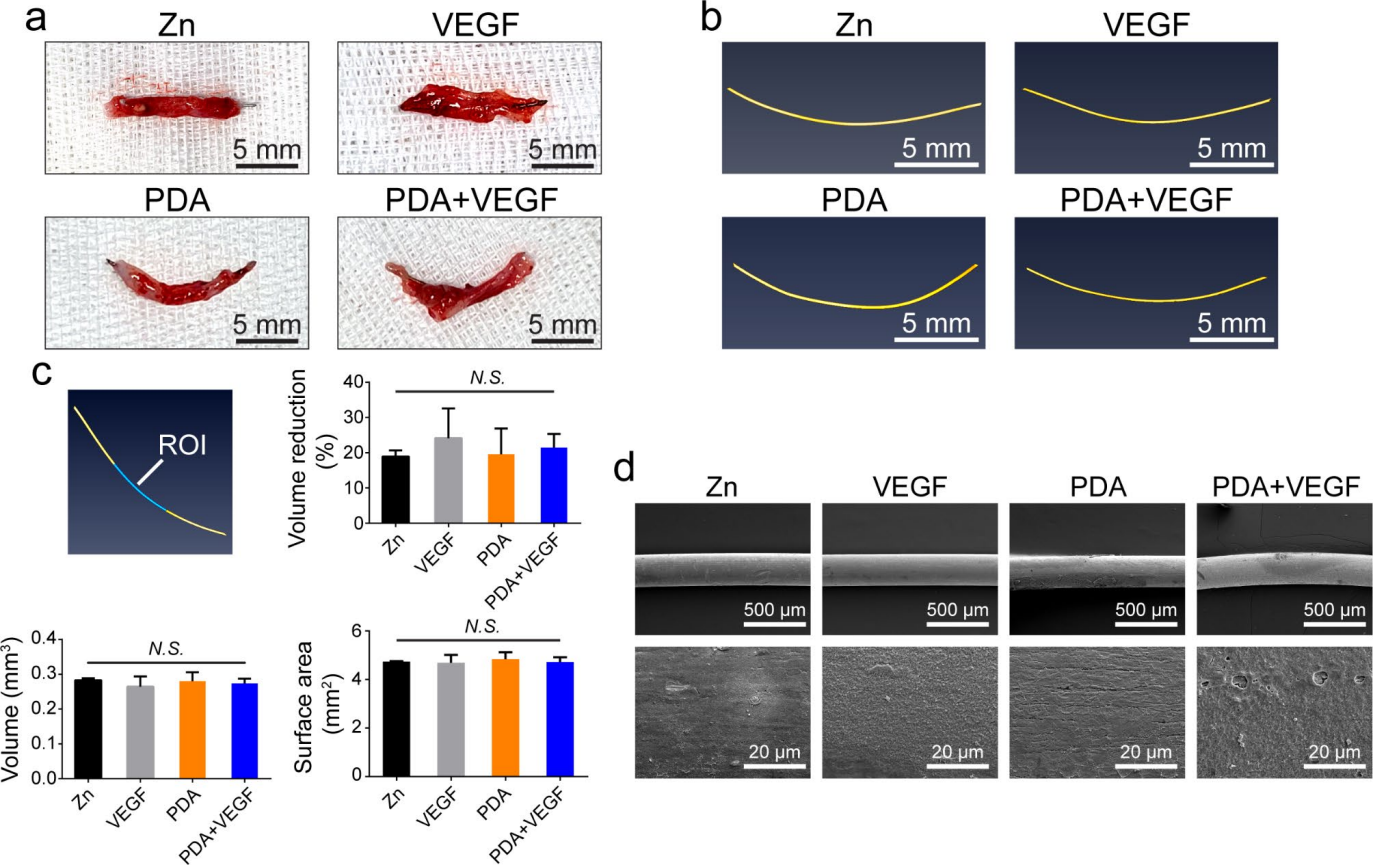
The degradation of different Zn wires in vivo. (**a**) The macroscopic views of the explanted Zn from the different groups. (**b**) The images of the different Zn wires by micro-CT scanning. (**c**) The volume and surface area of the different Zn wires implanted in the rat aortas. (**d**) SEM images of the different Zn wires implanted in the rat aortas

Zn as a new material for bioabsorbable stents has desired degradation rate in vivo [2]. To investigate whether PDA and VEGF coatings would affect the degradation of Zn, the Zn wires post implantation were scanned with micro-CT. Macroscopic views of the explanted Zn wires showed that the middle parts of the wires were inside the aortas and both ends were outside (Fig. 6a). Micro-CT scanning showed that there were no obvious degradation signs in any group (Fig. 6b). The analysis of the middle part of the wires (region of interests, ROI) exhibited that compared to the volumes of the Zn wires before implantation, there was 18.91%, 24.15%, 19.53%, and 21.43% reduction in the volume of the Zn wires in the Zn, VEGF, PDA and PDA + VEGF groups, respectively, and there was no significant difference among the different groups. Since the coated Zn wires lost similar volumes as the pure Zn wires within 1 month, the coated Zn wires had a degradation rate comparable to the pure Zn wires. Besides, there was no significant difference either in volume or surface areas among the different groups (Fig. 6c). SEM examination also confirmed these data (Fig. 6d). The VEGF-, PDA- and PDA + VEGF-coated Zn wires had relatively smooth surfaces, which were comparable to those of the pure Zn wires. These results indicate that the PDA-based coatings didn’t affect the degradation of Zn wires in vivo.

### Intimal hyperplasia surrounding PDA and VEGF-coated Zn wires in vivo

**Fig. 7:**
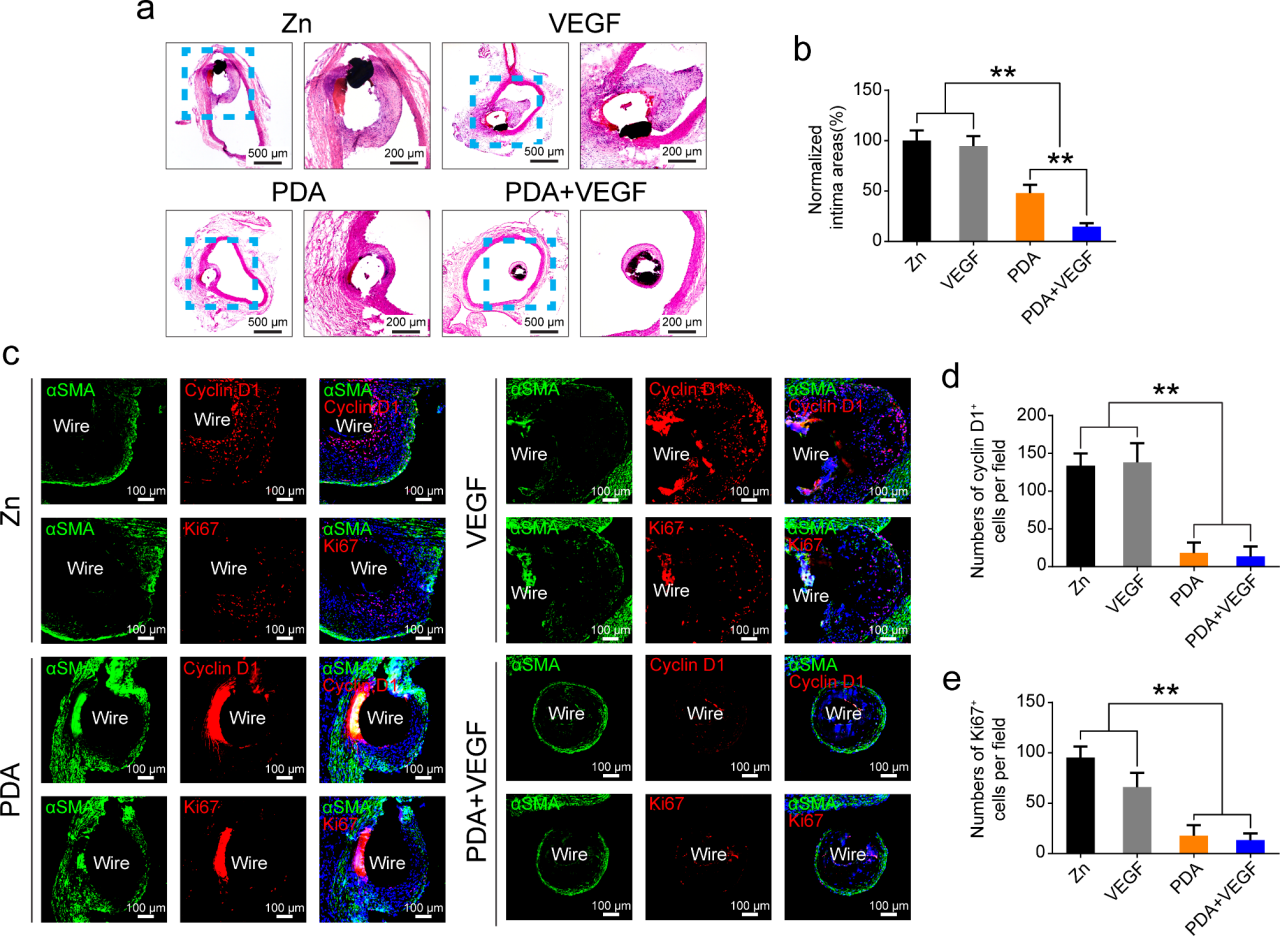
Intimal hyperplasia surrounding different Zn wires after aortic implantation. (**a**) H&E staining of the different Zn wires implanted in the rat aortas for 1 month. (**b**) Quantification of the areas of the neointimas of the different Zn wires. (**c**) Co-staining of the different Zn wires implanted in the rat aortas for 1 month with Cyclin D1and αSMA or Ki67 and αSMA. Quantification of Cyclin D1 positive cells (**d**) or Ki67 positive cells in the neointimas of the different Zn wires. * indicates p < 0.05, compared between two groups; ** indicates p < 0.01, compared between two groups

As shown in Fig. 7a, vascular responses to Zn wires in different groups differed dramatically. Both non-coated and VEGF-coated Zn wires had a thick layer of neointimas, while PDA-coated Zn wires had a thinner one. PDA and VEGF coatings further decreased the neointimal formation. Quantification data showed that the neointima in the PDA group was only 50% thick as compared to the pure Zn group and the neointima thickness in the PDA and VEGF group was only 14.57% (Fig. 7b). Immunofluorescence staining revealed that there were quite a lot of Cyclin D1^+^ or Ki67^+^ cells in the neointimas of the non-coated and VEGF-coated Zn wires, whereas such positive cells were rather few in the neointimas of the PDA-coated and PDA and VEGF-coated Zn wires (Fig. 7c). Quantification of the Cyclin D1^+^ or the Ki67^+^ cells demonstrated a similar trend, with more Cyclin D1^+^ or Ki67^+^ cells in the Zn and VEGF groups than those in the PDA and PDA + VEGF groups (Fig. 7d **and e**).

### EC coverage on PDA and VEGF-coated Zn wires in vivo

**Fig. 8:**
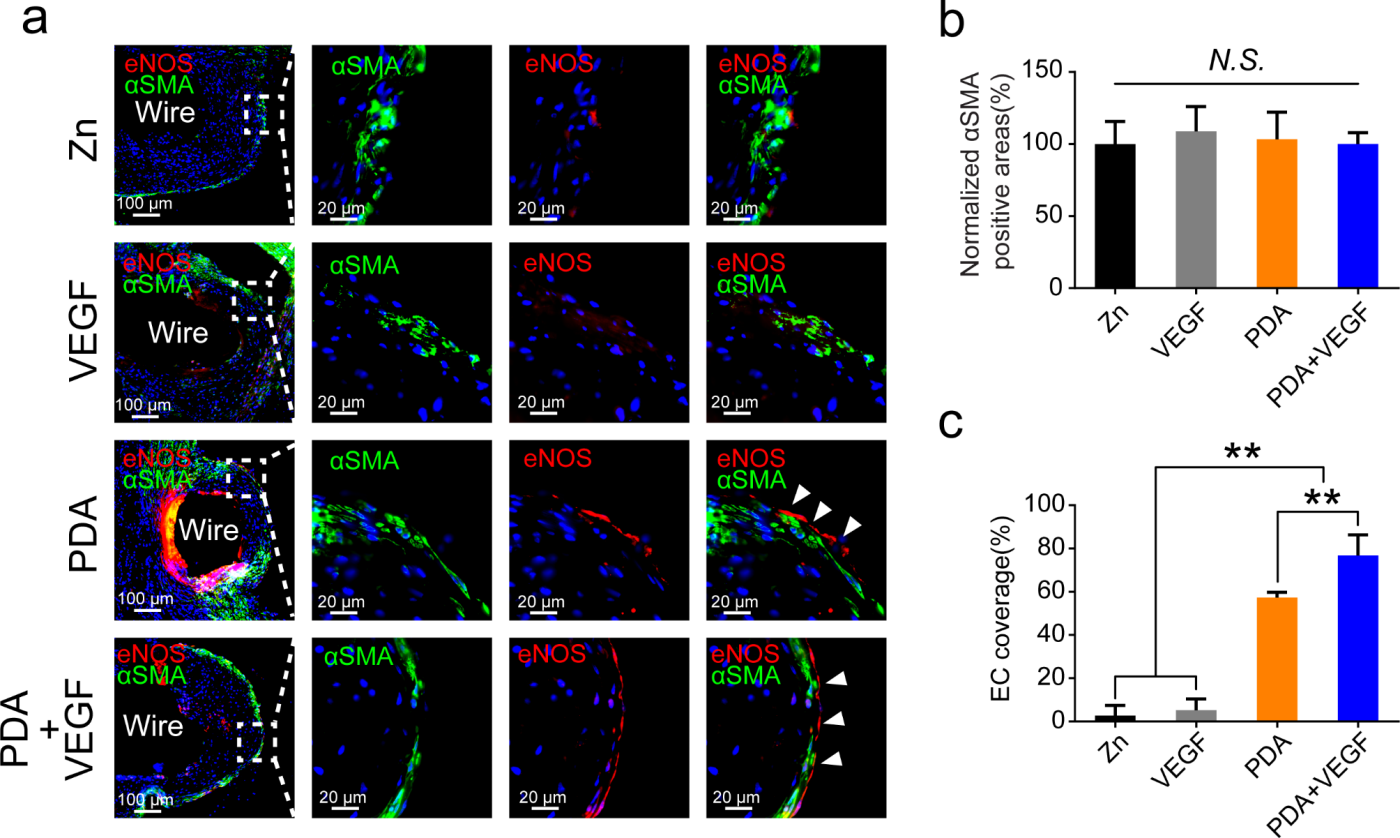
EC coverage of different Zn wires after aortic implantation. (**a**) Co-staining of the different Zn wires implanted in the rat aortas for 1 month with eNOS and αSMA. (**b**) Quantification of αSMA positive areas in the neointimas of the different Zn wires. (**c**) EC coverage of the different Zn wires implanted in the rat aortas for 1 month. * indicates p < 0.05, compared between two groups; ** indicates p < 0.01, compared between two groups

Rapid endothelization can efficiently prevent thrombosis after stent implantation in blood vessels. Therefore, EC coverage on Zn wires post implantation was evaluated in this study. Immunofluorescence staining showed that outer layers of neointimas consisted of smooth muscle cells (SMCs, αSMA^+^ cells) and endothelial cells (ECs, eNOS^+^ cells, Fig. 8a). There was no difference in αSMA positive areas among different groups (Fig. 8b). However, PDA coatings and PDA and VEGF coatings significantly increased percentage of the EC coverage from 2.73% in the pure Zn group and 5.29% in the VEGF group to 57.37% and 76.78%, respectively (Fig. 8a **and c**). These results demonstrate that PDA coatings can improve biocompatibility of Zn to ECs and accelerate the endothelization in vivo. Immobilization of VEGF on PDA coatings can further enhance the biocompatibility of Zn.

### Macrophage infiltration in neointimas of PDA and VEGF-coated Zn wires in vivo

**Fig. 9:**
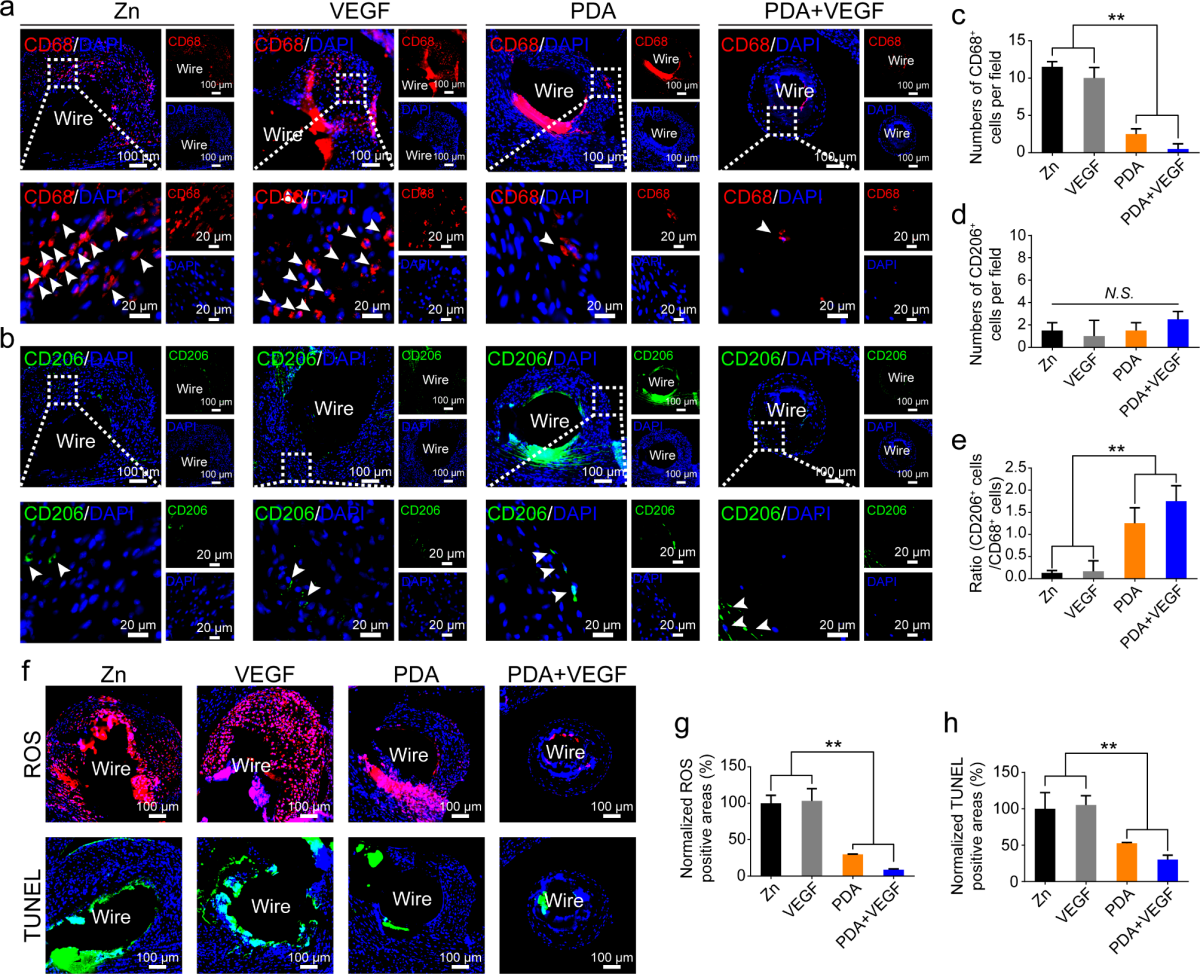
Macrophage infiltration in the neointimas of the different Zn wires after aortic implantation. (**a** and **b**) Staining of the different Zn wires implanted in the rat aortas for 1 month with CD68 or CD206. (**c-e**) Quantification of CD68^+^ cells, CD206^+^ cells and the ratio of the CD206^+^ cells to the CD68^+^ cells in the neointimas of the different Zn wires. (**f**) Staining of the different Zn wires implanted in the rat aortas for 1 month with ROS probes or TUNEL. Quantification of areas positive for ROS (**g**) or TUNEL (**h**). * indicates p < 0.05, compared between two groups; ** indicates p < 0.01, compared between two groups

Besides SMCs and ECs, we also evaluated macrophage infiltration in neointimas surrounding Zn wires in different groups. CD68 was used as a common marker for macrophages and CD206 was used as a marker for M2 macrophages. Both non-coated and VEGF-coated Zn wires had infiltration of numerous CD68 positive cells (Fig. 9a). In contrast, the PDA group had limited infiltration of CD68 positive cells, and the PDA and VEGF group had even fewer CD68 positive cells. Quantification data also confirmed this result, with significantly fewer CD68 positive cells in the PDA and PDA + VEGF groups than those in the Zn and VEGF groups (Fig. 9c). As shown in Fig. 9b, the CD206^+^ cells were quite few in all the groups. However, the ratios of CD206^+^ cells to CD68^+^ cells in the PDA + VEGF and PDA groups were higher than those in the Zn and VEGF groups (Fig. 9e). The high ratios of CD206^+^ cells to CD68^+^ cells probably contribute to the reduced inflammation responses. Meanwhile, the Zn and VEGF groups showed significantly higher ROS levels than the PDA and PDA + VEGF groups (Fig. 9f **and g**). TUNEL staining also showed more cell apoptosis in the Zn and VEGF groups than that in the PDA and PDA + VEGF groups (Fig. 9f **and h**). The improved biocompatibility of Zn wires after PDA coating induced less macrophage infiltration and thus low levels of ROS and cell apoptosis.

### Extracellular matrix (ECM) remodeling surrounding PDA and VEGF-coated Zn wires in vivo

**Fig. 10:**
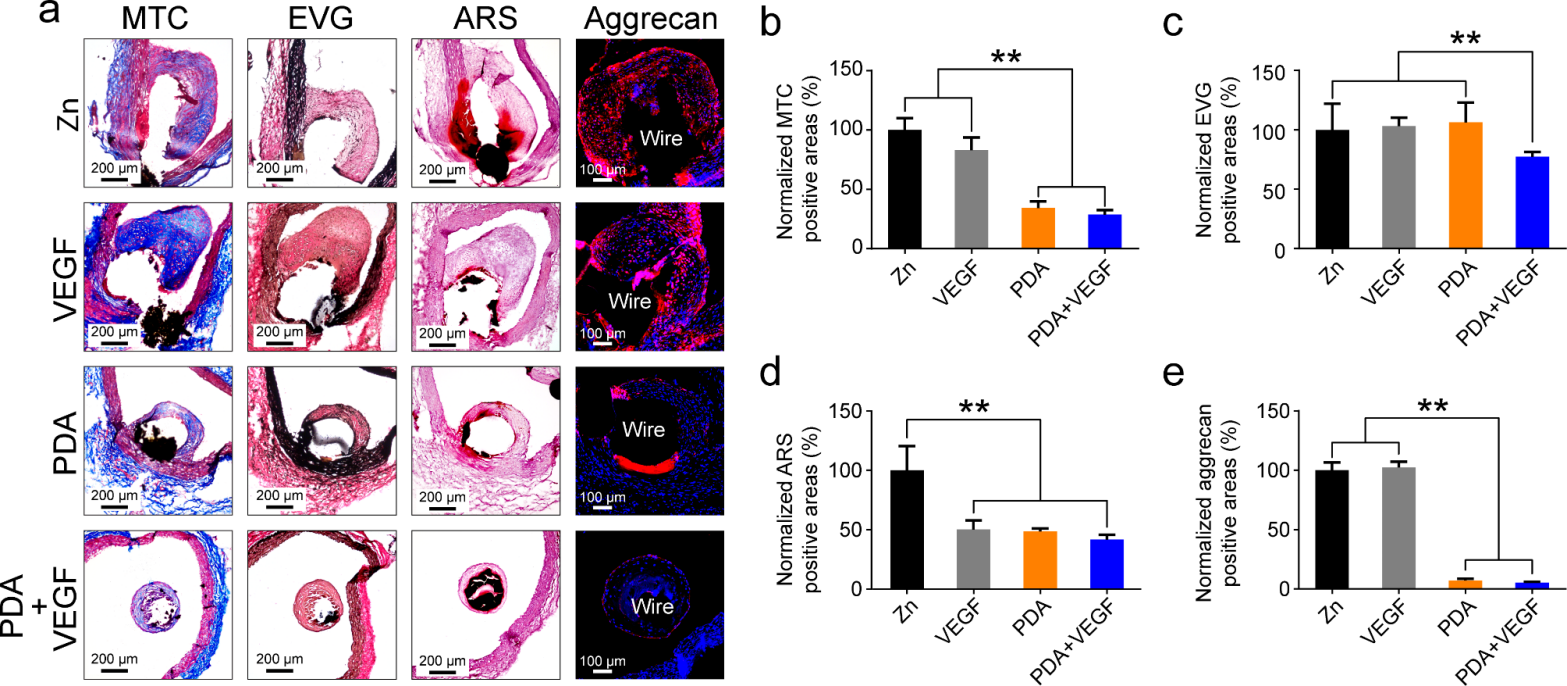
ECM in the neointimas of the different Zn wires after aortic implantation. (**a**) Staining of the different Zn wires implanted in the rat aortas for 1 month with MTC, EVG, ARS, and aggrecan. (**b**) Quantification of MTC positive areas (**b**), EVG positive areas (**c**), ARS positive areas (**d**), and aggrecan positive areas (**e**) in the neointimas of the different Zn wires. * indicates p < 0.05, compared between two groups; ** indicates p < 0.01, compared between two groups

Finally, we characterize ECM components of neointimas surrounding Zn wires. The MTC staining showed that collagens were the main component of the ECM (Fig. 10a). The Zn and VEGF groups had higher collagen contents than the PDA and PDA + VEGF groups did (Fig. 10b). Elastin was less expressed in the neointimas surrounding Zn wires as compared to the collagens. Besides, the level of elastin in the PDA + VEGF group was lower than any other three groups (Fig. 10c). The ARS staining showed obvious calcification in the Zn group, whereas the other three groups had no obvious calcification (Fig. 10a **and d**). An increase in aggrecan expression is indicative of osteoblast-like differentiation of SMCs [30, 31]. The neointimas surrounding the pure Zn wires and the Zn wires coated with VEGF were positively stained for aggrecan, whereas the PDA and PDA + VEGF groups had quit few aggrecan-positive areas (Fig. 10a). Compared to the pure Zn wires, the neointimas in the PDA and PDA + VEGF groups had only 7.14% and 5.24% aggrecan-positive areas of the Zn group, respectively (Fig. 10e).

## Discussion

Inspired by compositions of adhesive plaques of mussels, self-polymerization of dopamine, containing both catechol and amine groups, is utilized to form a thin and adherent layer of PDA coatings on diverse substrate surface [16]. Under alkaline conditions, surface-tethered catechol groups shift toward the quinone groups, holding a latent reactivity toward nucleophiles, such as amine groups of proteins, for bioconjugation reactions [17]. Bioactive molecules then can be immobilized onto the PDA coatings through covalent binding to create a bioactive surface. Consequently, compared to the non-PDA-treated Zn surfaces, the PDA-coated Zn surfaces exhibited more BSA immobilization (Fig. 2), although there was a dramatic decline in the BSA fluorescence intensity on both PDA-coated Zn surfaces and the pure Zn surfaces on day 3 (Fig. 2e **and f**). This observation is probably due to the quenching of the RBITC that was used to label the BSA. On the other hand, since endothelial cells can efficiently inhibit thrombosis and neointimal formation [32, 33], we thus chose VEGF as ad-layers to promote attachment, spreading, proliferation, and phenotype maintenance of ECs on Zn surfaces. Indeed, ECs had optimal behaviors on the PDA and VEGF-coated Zn surfaces (Figs. 3 and 4), which is consistent with previous studies [24, 25]. However, in contrast to cobble-like structures of ECs on substrates of stainless steels and titanium, ECs displayed a round morphology even on the PDA and VEGF coated Zn surfaces, probably due to a high concentration of Zn^2+^ released into cell culture media (over 10 µg/ml/day) during Zn degrades [13, 15, 34]. Generally, Zn degrades 2–3 times faster under in vitro conditions than in vivo ones [5]. Because of a lower concentration of Zn^2+^ released during slower degradation of Zn in vivo, Zn usually exhibits a better biocompatibility in vivo scenario.

In this study, a wire aortic implantation model in rats was used to evaluate vascular responses of surface-modified Zn in vivo, which allows a reliable study of hemocompatibility, biodegradation, and intima hyperplasia of materials for stents under a real vascular environment, including the blood- and vascular wall-material interfaces (Fig. 5a) [2, 5, 9, 12, 35–37]. The diameter of the Zn wires used in this study was around 125 μm, falling within the range of structs of traditional metal stents (60–140 μm, made from stainless steels or cobalt-chromium alloy) [38]. Therefore, the interfaces between the Zn wires and blood can well represent those between stents and blood, although the Zn wires don’t assume mechanical stress that stents are subjected to when deployed in coronary arteries [5]. In addition, due to real blood-material interfaces created in this model, hemocompatibility of materials or coatings for stents can be precisely evaluated via ultrasound imaging (Fig. 5b-f). Similarly, degradation of materials for stents in blood can also be accurately assessed with micro-CT and SEM (Fig. 6). Moreover, the wire aortic implantation model provides us an opportunity to observe intimal hyperplasia surrounding materials for stents in vivo (Fig. 7). Intimal hyperplasia is the main reason for in-stent restenosis and limited intimal hyperplasia post stent implantation is a golden standard to judge whether stents have good biocompatibility [1]. Because of complicated mechanisms for intimal hyperplasia, it will be difficult to recapitulate intimal hyperplasia in vitro and judge biocompatibility of stents only through in vitro experiments. Therefore, the wire aortic implantation model used in this study is a valuable platform to evaluate new materials or coatings for stents in vivo which can greatly reduce cost for new stent development. Of course, after screening out candidate materials or coatings for stents, real stent manufacturing and coronary artery implantation in large animals, such as pigs, are still needed before clinical trials.

In this study, we found that the PDA coatings alone could greatly improve biocompatibility of Zn to HUVECs in vitro as compared to the non-coated or VEGF-coated Zn (Figs. 3 and 4). These results are consistent with previous studies which show that PDA can effectively improve EC attachment, proliferation, and migration because of rich amino groups in PDA [39, 40]. Deposition of VEGF on PDA layers further improved the biocompatibility of Zn, probably because a more favorable microenvironment was constructed on Zn surfaces by VEGF, which play an important role in vascular development [41]. Accordingly, in vivo data supported the improved hemocompatibility, reduced neointimal formation, increased EC coverage, decreased macrophage infiltration, and declined aggrecan accumulation in ECM after PDA coating (Fig. 5, and Figs. 7, 8, 9 and 10). We also observed a decreased ROS level in the neointimas surrounding the Zn wires coated with PDA, probably because abundant phenol groups in PDA coatings can eliminate ROS as a radical scavenger [19]. Our findings, for the first time, highlight that PDA coatings alone increase hemocompatibility of Zn and decrease neointimal formation post Zn wire implantation in vivo. Although PDA and VEGF coatings further inhibited intimal hyperplasia possibly due to an accelerated reendothelialization in vivo (Fig. 7), but the improvement is limited. Considering the high costs of VEGF for commercial use, PDA is probably a better choice due to its lower price and simplicity in coating diverse materials. Of course, optimization of PDA coatings for Zn is still needed to achieve minimal neointimal hyperplasia after post Zn implantation in vivo.

Although the use of PDA coatings alone to improve biocompatibility of biomaterials or as ad-layers to immobilize growth factors to further enhance the biocompatibility of biomaterials has been widely reported, whether this strategy can improve biocompatibility of Zn as materials for vascular stents has not been tested yet. For instance, PDA coatings alone or further immobilization of VEGF on the PDA coatings can enhance phenotypes of ECs on titanium; the immobilized VEGF can even promote differentiation of mesenchymal stem cells into ECs [25, 42]. Besides, immobilization of VEGF via PDA coatings on PLCL elastomers can support EC behaviors [43]. These studies indicate that immobilization of VEGF on substrates via PDA coatings is an effective approach to encourage EC growth. However, these studies are performed in vitro, and the effect of the PDA coating combined with VEGF is not validated in vivo. In addition, whether the utilization of PDA coatings can immobilize VEGF on Zn substrates and the effect of this coating on Zn substrates are not tested in these studies. In addition, Sun et al. showed that VEGF and bone morphogenetic protein-2 (BMP-2) could be immobilized onto poly-L-lactic acid (PLLA) nanofibers via PDA coatings. The PDA coatings increased the loading capacity of VEGF and BMP-2 and achieved a slow and sustained release of them. The in vivo data showed that the immobilization of BMP-2 and VEGF on PLLA nanofibers via PDA coatings enhanced the vascular formation and new bone formation in a rat femoral defect model [44]. Huang et al. showed that immobilization of VEGF on allogenic bone grafts via PDA coatings improved bone regeneration in a rabbit bone defect model after 12 weeks of implantation [45]. Lee et al. immobilized VEGF on polycaprolactone (PCL) vascular grafts via PDA coatings, which elicited enhanced proliferation and angiogenic differentiation of vascular cells [29]. These studies demonstrate the efficacy of VEGF immobilization via PDA coatings in promoting vascular formation in vivo. However, whether the same strategy can accelerate endothelization on Zn substrates is unknown. A more relevant in vivo study showed that PDA coatings could reduce in-stent restenosis of cobalt-chromium (CoCr) stents implanted in rat aortas [28]. CoCr is a non-degradable metal, while Zn is a bio-degradable one. Although the PDA coatings can inhibit intimal hyperplasia of CoCr stents, the effect of the PDA coatings on intimal hyperplasia of Zn still needs to be validated. Furthermore, whether VEGF immobilization via PDA coatings can further enhance the effectiveness of the PDA coatings is not investigated in that study. In this study, we first coated the Zn substrates with PDA and then immobilized VEGF onto the PDA coatings. Adhesion, proliferation, and phenotype maintenance of ECs on the coated Zn were evaluated in vitro. Then, a wire aortic implantation model in rats mimicking endovascular stent implantation in humans was used to assess vascular responses to the coated Zn wires in vivo. Indeed, a similar strategy has been reported previously by other groups. However, the effects of this coating on biodegradable Zn substrates haven’t been reported yet. In addition, our current study focuses on the in vivo applicability of this coating and reveals the impacts of this coating on Zn degradation and vascular responses from a unique perspective.

## Conclusion

In this study, we coated Zn, a new material for bioabsorbable stents, with PDA and VEGF to increase its biocompatibility. PDA-coated Zn showed an increased EC adhesion, spreading, proliferation, and phenotype maintenance on its surfaces. VEGF immobilization onto PDA coatings further enhanced the biocompatibility of Zn. Implantation of PDA-coated Zn into rat aortas showed an improved hemocompatibility, reduced neointimal formation, increased EC coverage, decreased macrophage infiltration, and declined aggrecan accumulation in ECM. VEGF and PDA coatings further increased the EC coverage as compared to the PDA coatings alone and hence resulted in thinner neointimas. These results indicate that functionalization of Zn surfaces with PDA and VEGF is a promising strategy to inhibit neointimal formation post Zn implantation in vivo.

## Data Availability

All data generated or analysed during this study are included in this published article.

## List of abbreviations

SMC: Smooth muscle cell
Zn: Zinc
PDA: Polydopamine
PDMS: Poly(dimethylsiloxane)
PU: Poly(urethane)
EC: Endothelial cell
ECM: Extracellular matrix
RBITC: Rhodamine B isothiocyanate
SEM: Scanning electron microscope
IVIS: In Vivo Imaging System
CCK-8: cell counting kit-8
PFA: Paraformaldehyde
DAF-FM DA: 3-Amino,4-aminomethyl-2’,7’-difluorescein diacetate
BSA: Bovine serum albumin
eNOS: Endothelial nitric oxide synthase antibody
VWF: Von Willebrand Factor antibody
VEGF: Vascular endothelial growth factor
VTI: Velocity time integral
Micro-CT: Micro-computed tomography
3D: 3-dimensional
OCT: Optimal cutting temperature compound
H&E: Hematoxylin and eosin
MTC: Masson’s trichrome
EVG: Elastic Verhoeff-Van Gieson
ARS: Alizarin red S
TUNEL: Terminal deoxynucleotidyl transferase-mediated dUTP nick end labeling
ROS: reactive oxygen species
αSMA: Alpha smooth muscle actin antibody
HUVEC: Human umbilical vein endothelial cell
PBS: phosphate buffered saline
